# Prevalence of multidrug resistance in *Pseudomonas* spp. isolated from wild bird feces in an urban aquatic environment

**DOI:** 10.1002/ece3.8146

**Published:** 2021-09-21

**Authors:** Joana G. C. Rodrigues, Harisree P. Nair, Christopher O'Kane, Caray A. Walker

**Affiliations:** ^1^ School of Life Sciences Faculty of Science & Engineering Anglia Ruskin University Cambridge UK

**Keywords:** antimicrobial resistance, epidemiological studies, one health, *Pseudomonas* spp., wild birds

## Abstract

Antimicrobial resistance (AMR) has been detected in the microbiota of wildlife, yet little is known about the origin and impact within the ecosystem. Due to the shortage of nonepizootic surveillance, there is limited understanding of the natural prevalence and circulation of AMR bacteria in the wild animal population, including avian species. In this surveillance study, feces from wild birds in proximity to the River Cam, Cambridge, England, were collected and *Pseudomonas* spp. were isolated. Of the 115 samples collected, 24 (20.9%; 95% CI, 12.6%‒29.2%) harbored *Pseudomonas* spp. of which 18 (75%; 95% CI, 58%‒92%) had a multiple antibiotic resistance (MAR) index greater than 0.2. No *Pseudomonas* spp. isolate in this study was pansusceptible. Resistance was found among the 24 isolates against ciprofloxacin (87.5%; 95% CI, 74.3%‒100%) and cefepime (83.3%; 95% CI, 68.4%‒98.2%), both of which are extensively used to treat opportunistic *Pseudomonas* spp. infections. The prevalence of *Pseudomonas* spp. in the wild bird feces sampled during this study is greater than previous, similar studies. Additionally, their multidrug resistance profile provides insight into the potential risk for ecosystem contamination. It further highlights the importance of a One Health approach, including ongoing surveillance efforts that help to develop the understanding of how wildlife, including avifauna, may contribute and disperse AMR across the ecosystem.

## Graphical abstract

Wild bird fecal samples were collected from urban areas surrounding the River Cam. A 20.9% Pseudomonas spp. prevalence rate was found in a total of 115 faecal samples. The majority (75%) of the isolates were resistant to more than one antibiotic tested.

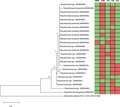

## 1 INTRODUCTION

More than 60% of human infections each year are attributable to zoonotic diseases (Karesh et al., 2012). It is therefore essential to identify and monitor potential pathogen reservoirs, including birds, whose mobility and heterogeneous environmental exposure may facilitate pathogen spillover. The emergence of infectious diseases in wildlife and their potential threat as zoonoses has resulted in increased research interest in birds as sentinels, vectors, and spillover sources of antimicrobial resistance (AMR) (Dolejska & Papagiannitsis, 2018). Zoonoses are of growing concern due to the impact of the ever‐increasing human population and ecosystem changes that bring people and wildlife into closer and more frequent contact (Laurance et al., 2014; Muehlenbein, 2013; Nadimpalli et al., 2020).

Despite AMR being commonly associated with high antibiotic usage, this is generally not true in wildlife; therefore, its presence may be used as an indicator of anthropogenic activities affecting the whole ecosystem. For instance, sewage water treatment facilities, farm manure, and slurry are all important habitats for birds and other animals, but they can become contaminated with AMR bacteria, antibiotics (and/or their metabolites), and other elements that can act as selective drivers of AMR. Although assigning the directionality of this dissemination process is an extremely challenging task, previous work has noted the association between resistance patterns and the physical proximity of wild animals to humans (Laurance et al., 2014; Muehlenbein, 2013; Nadimpalli et al., 2020). Wild birds in particular may act as vectors of AMR bacteria by acquiring them from contaminated environments such as rivers receiving sewage effluent and, subsequently, contaminating other environments such as livestock grazing areas and urban environments through fecal shedding. This process potentially facilitates the dissemination of enteric pathogens of public health concern including, but not limited to, *Pseudomonas* spp., *Salmonella* spp., *Klebsiella* spp., *Campylobacter* spp., and *Staphylococcus aureus* (Benskin et al., 2009; Navarro‐Gonzalez et al., 2020).

The *Pseudomonas* genus contains over 60 species of Gram‐negative, aerobic, nonspore forming, rod‐shaped, and motile organisms. *Pseudomonas* spp. are also capable of protecting other organisms by sheltering them from unfavorable conditions within biofilm formations (Puga et al., 2018) as well as employing cooperation mechanisms such as quorum sensing (Venturi et al., 2010). *Pseudomonas* are a metabolically versatile genus with typically large genome sizes varying from 3 to 7 Mbp (Hesse et al., 2018), known to contain several genetic mobile elements including megaplasmids (Cazares et al., 2020) as well as intrinsically and extrinsically acquired resistance mechanisms (Lister et al., 2009). These biological properties allow *Pseudomonas* spp. to survive in a multitude of environments, including community reservoirs such as soil and rhizosphere, swimming pools, and other infrastructures within urban settings (Nadimpalli et al., 2020). Carbapenems, cephalosporins, fluoroquinolones, and aminoglycosides are the most frequently used antipseudomonal antibiotics but resistance, including multidrug and pandrug resistance, has been reported in both veterinary and human medicine (Cabassi et al., 2017; Cazares et al., 2020; Haenni et al., 2015, 2017). The resistance mechanisms used by *Pseudomonas* spp. are varied and facilitated by their genomic plasticity including multidrug efflux systems, outer membrane protein loss, target mutations, and enzyme production (Cabassi et al., 2017).

One species within the *Pseudomonas* genus, *Pseudomonas aeruginosa*, is the causative agent of several diseases ranging from external otitis to fatal pneumonia in a range of hosts including dogs, rabbits, birds, and humans. It is rarely a member of the normal microbial flora in healthy humans or animals (Lister et al., 2009). However, in humans, severe *P. aeruginosa* infections usually occur in immunocompromised patients and in healthcare settings. In the United Kingdom, between April 2019 and March 2020, *P. aeruginosa* was the second most frequent nosocomial infection with 24.8% of all reported cases (*n* = 4,336) leading to death (Public Health England, 2021). Other species in the *Pseudomonas* genus include prolific plant and aquaculture pathogens (Beaton et al., 2018), food spoilage‐associated organisms (Stellato et al., 2017), and useful biocontrol agents against plant pathogens (Gómez‐Lama Cabanás et al., 2018; Haas & Défago, 2005; Kuzmanović et al., 2018).

Previous studies exploring the natural prevalence of bacteria associated with birds investigated microorganisms that present a threat to either human or domesticated/production animal health (Haesendonck et al., 2016; Laurance et al., 2014; Navarro‐Gonzalez et al., 2020). The majority of these studies provide little information on the nonepizootic prevalence of *Pseudomonas* spp. in avifauna as they tend to rely on *postmortem* examinations and/or data collected as a result of a disease outbreak causing high mortality (Gómez, 2006; Vasconcelos et al., 2017; Vidal et al., 2017; Walker et al., 2002). Ongoing AMR surveillance studies of *Pseudomonas* spp. in wildlife, including birds, represent an underexplored area that may pose risks to humans, other animals, and the environment (Hernando‐Amado et al., 2019; O'Neill, 2016; Pornsukarom & Thakur, 2017). The present study reports the nonepizootic prevalence of *Pseudomonas* spp. in feces from wild birds in an urban aquatic setting. Additionally, the isolates' antimicrobial susceptibility was determined in order to estimate potential risks to other elements of the ecosystem, which is the overarching purpose of this research.

## 2 MATERIALS AND METHODS

### 2.1 Fecal sampling

**FIGURE 1:**
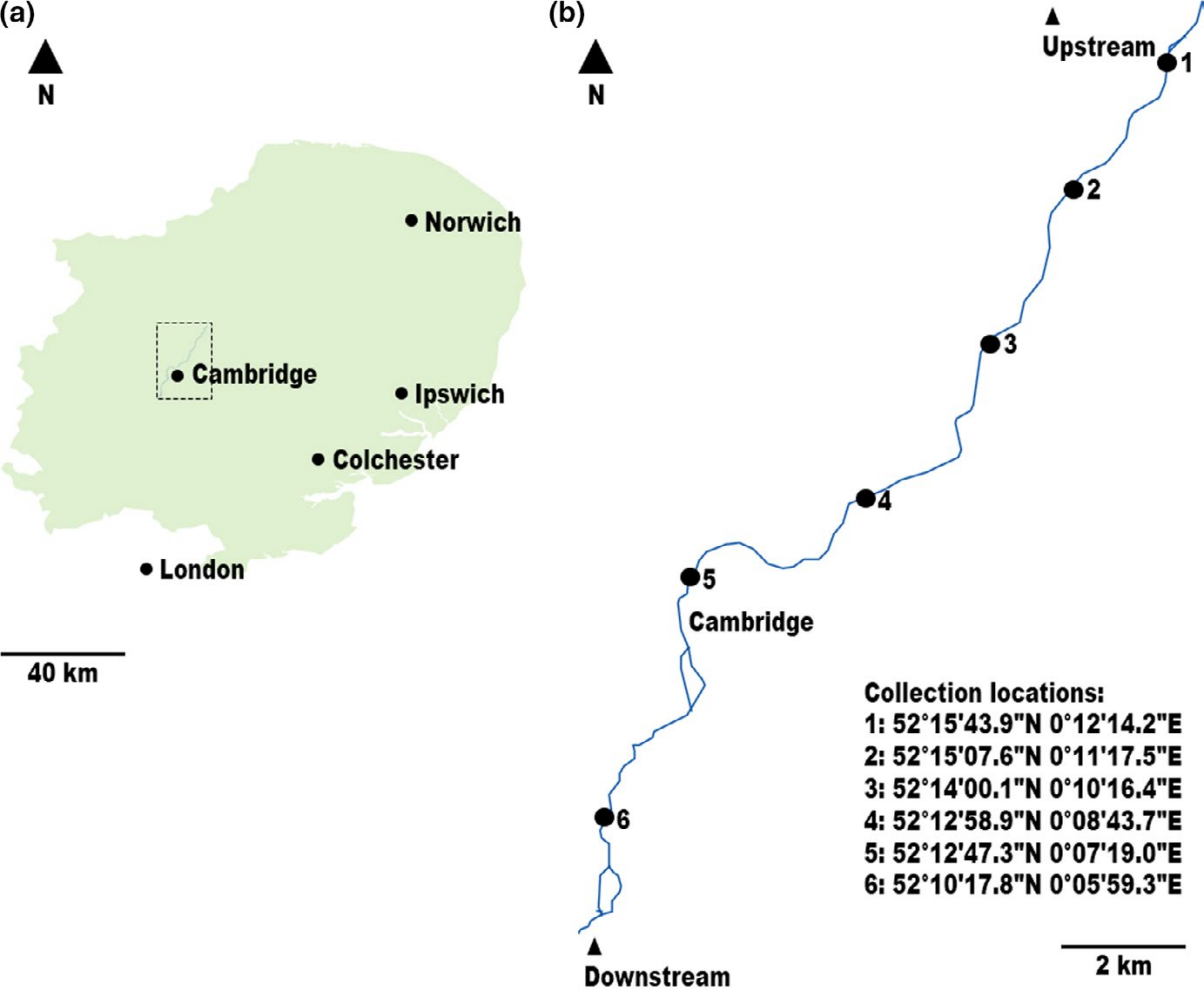
(a) Schematic map of East Anglia as legally defined in the Nomenclature of Territorial Units for Statistics (NUTS) 2. The map illustrates key geographic locations surrounding the sample collection area (delineated by the dotted square); Scale bar, 40 km. (b) Trace map of the River Cam, Cambridge, alongside the geographic coordinates of each sample collection site (expressed as latitude and longitude, in degrees, minutes, and seconds, according to the global positioning system); Scale bar, 2 km

A total of 115 bird fecal samples were collected using Amies Plain Transwabs^®^ (MWE, Wiltshire). Swabs were transported to a containment level 2 laboratory where they were stored at 4°C for up to 24 hr before culturing. The bird fecal swabs were collected over a 24‐month period from 6 locations along a 16‐km stretch of the River Cam, Cambridge, England, and up to 0.8 km away from the riverbank (Figure 1). The sampled locations include leisure locations (e.g., nature reserve, country park, rowing club) that are popular with human pursuits such as boating, swimming, and bird‐watching. Although swabs were obtained regularly from each location throughout the sampling period (at least twice), samples were collected during all seasons while maintaining a minimum dry weather timespan of 48 hr to avoid rainfall‐associated fecal microbiota changes (Shehane et al., 2005). Care was taken to sample freshly defecated specimens from areas where Passeriformes, Columbiformes, Anseriformes, and Charadriiformes were observed to be inhabiting/visiting (Shehane et al., 2005).

### 2.2 Sample culture and isolation of *Pseudomonas* spp.

Within 24 hr of collection, the bird fecal swabs were streaked onto *Pseudomonas* cetrimide agar (Oxoid, Basingstoke) and incubated aerobically at 37°C for 24 hr. Plates with confluent growth were subcultured for isolation to avoid the risk of competitive inhibition. Putative *Pseudomonas* spp. colonies from the primary isolation media were subcultured, and isolated colonies were subject to Gram staining followed by biochemical testing for cytochrome c oxidase production and lactose fermentation via commonly used microbiological techniques (Public Health England, 2015). At least three passages were done and tested as described to obtain pure cultures.

### 2.3 Bacterial identification by 16S rRNA gene sequencing

Once pure cultures of bacteria were isolated and established, DNA was extracted using DNeasy Blood & Tissue Kit (Qiagen) as per the manufacturer's instructions. The DNA of individual putative *Pseudomonas* spp. isolates was used as a template to amplify the region encoding the 16S rRNA gene (1.5 kb) in a thermal cycler (Techne, Loughborough) using previously described primers complementary to the conserved regions of the 5′ and 3′ ends of 16S rRNA gene (Lane et al., 1991). A reaction mixture consisting of 1× MyTaq™ Red Mix (Bioline, London), 1 μl of template DNA (50–100 ng), and 0.5 μM of each primer in a final reaction volume of 25 μl was used. PCR conditions were as follows: 95°C for 5 min, 34 cycles of 30 s at 95°C, 30 s at 54°C, and 2 min at 72°C were carried out followed by a terminal elongation step at 72°C for 10 min. The amplicons were separated electrophoretically through a 1% agarose gel in 1× TAE buffer and visualized using GelRed^®^ Nucleic Acid Gel Stain (Biotium, California) under ultraviolet illumination. Gel images were captured using GeneSys (Syngene, Cambridge), and the products were identified by molecular weight comparison with the markers of a 1 kb Plus DNA ladder (Invitrogen, Loughborough). The amplified PCR products were purified using the QIAquick PCR Purification Kit (Qiagen, Germany) and partially sequenced at the Department of Biochemistry, University of Cambridge, UK (https://www.bioc.cam.ac.uk/), using the forward primer (Lane et al., 1991). The sequences' similarity were determined by comparing with the GenBank database using BLAST (Altschul et al., 1990).

To determine the evolutionary relatedness among the *Pseudomonas* spp. isolates obtained in this study, a phylogenetic analysis was performed, employing MEGA version X (Kumar et al., 2018). The sequences were compiled and aligned using ClustalW embedded in MEGAX. The phylogenetic tree was constructed using the Neighbor‐Joining method (Saitou & Nei, 1987), and evolutionary distances were inferred using the Tamura–Nei model (Tamura & Nei, 1993). The reliability of the tree was evaluated by bootstrap resampling technique with 1,000 bootstrap replications. *Salmonella enterica* ATCC 13314 (NR 041696) sequence retrieved from GenBank was used as an outgroup.

### 2.4 Antibiotic susceptibility testing

According to the European Committee on Antimicrobial Susceptibility Testing (EUCAST), antibiotic susceptibility testing was carried out using the disk diffusion method (The European Committee on Antimicrobial Susceptibility Testing, 2019). The tested antibiotics (Oxoid, Basingstoke) were meropenem (10 µg), cefepime (30 µg), gentamicin (30 µg), ciprofloxacin (5 µg), and levofloxacin (5 µg). After aerobic incubation at 37°C for 24 hr, the diameters of the zones of inhibition, in millimeters, were measured for each antibiotic and the isolates were classified as resistant or susceptible (The European Committee on Antimicrobial Susceptibility Testing, 2019). Isolates classed as “susceptible, increased exposure” by EUCAST were included in the susceptible category. Multiple antibiotic resistance (MAR) indices were then determined for each isolate by using the formula *MAR* = *a/b*, where *a* represents the number of antibiotics to which the isolate was resistant to and *b* represents the total number of antibiotics to which the isolate has been tested for susceptibility (Krumperman, 1983).

### 2.5 Statistical analysis

Statistical analysis was performed using SPSS Statistics 26 (IBM, Chicago). To investigate the differences in the number of isolates collected between the sampled locations, a chi‐square test was calculated. A chi‐square test was also performed to compare the number of resistant and susceptible isolates, for each antibiotic. In the sample collection locations where *Pseudomonas* spp. were isolated, a two‐way chi‐squared test was used to determine whether there was an association between the sample location and the resistance profile of the isolates obtained. Statistical significance was deemed as *α* = 0.05.

## 3 RESULTS

*Pseudomonas* spp. were detected in 24 of the 115 bird fecal samples collected and tested during this study (20.9%; 95% CI, 12.6%‒29.2% prevalence). Samples were collected from all six locations but were unevenly distributed across different sites (*p* < .001) (Table 1). The distribution of *Pseudomonas* spp. isolates was also significantly different between sample collection locations (*p* < .05), with one (location 3) yielding no isolates (Table 1).

**TABLE 1:** Distribution of *Pseudomonas* spp. isolates from wild bird feces across each sample collection location

Source	No. samples collected	No. *Pseudomonas* spp. isolates	% *Pseudomonas* spp. from samples collected	% MDR among *Pseudomonas* spp. isolates	Average MAR index
Location 1	8	3	38	100.0	0.53
Location 2	28	4	14	75.0	0.4
Location 3	6	0	0	–	–
Location 4	49	12	24	66.7	0.37
Location 5	20	4	20	75.0	0.40
Location 6	4	1	25	100.0	0.60
Total	115	24	20.9	75	0.41

Note

The findings are presented alongside the percentage of *Pseudomonas* spp. isolates that were resistant to more than one of the antibiotics tested and, therefore, multidrug resistant (MDR). The multiple antibiotic resistance (MAR) indices for each location were calculated as per Krumperman (1983). The antibiotics tested by disk diffusion were meropenem (10 μg), cefepime (30 μg), gentamicin (30 μg), ciprofloxacin (5 μg), and levofloxacin (5 μg).

**FIGURE 2:**
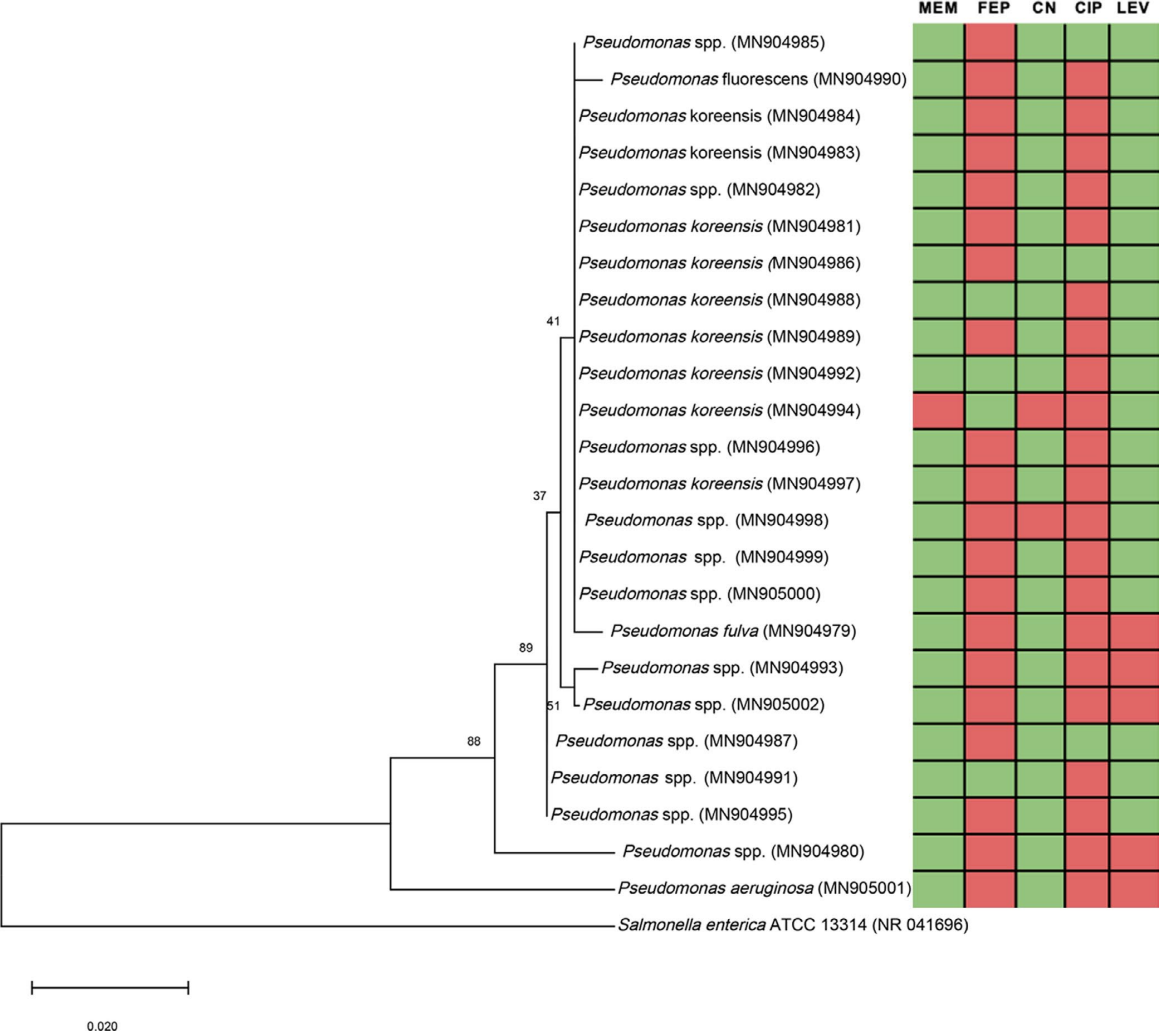
Phylogenetic tree of *Pseudomonas* spp. isolates from the study overlaid with a heatmap of the AMR profiles. The phylogenetic tree is based on 16S rRNA gene sequences of *Pseudomonas* spp. isolated from wild bird feces (*n* = 24) collected around the River Cam, Cambridge, England. GenBank accession numbers are given in parentheses. Values at branch nodes indicate the bootstrap values, and scale bar represents nucleotide substitutions per site. *Salmonella enterica* ATCC 13314 was used as an outgroup. The heatmap is color‐coded to denote resistance (in red) and susceptibility (in green) of each of the *Pseudomonas* spp. isolates. The antibiotics used are listed at the top of the heatmap by their standard abbreviation: CIP, ciprofloxacin; CN, gentamicin; FEP, cefepime; LEV, levofloxacin; MEM, meropenem

Of the 24 *Pseudomonas* spp. isolates, 12 exhibited a 16S rRNA gene similarity of <98.7% with any *Pseudomonas* spp. and, thus, were classified only to the genus level. Several isolates (*n* = 9) were identified as *P. koreensis*, and one isolate (*n* = 1) was identified as *P. aeruginosa*. The remaining isolates belonged to the *P. fulva* (*n* = 1) and *P. fluorescens* (*n* = 1) species. Based on the phylogenetic analysis, a monophyletic tree was obtained (Figure 2) with the sequences grouped within two major clades, which were distantly separated from the outgroup (*Salmonella enterica* ATCC 13314). *P. aeruginosa* formed a single clade, with all the remaining sequences clustered separately with different subclades.

Antibiotic sensitivity screening revealed that the majority of *Pseudomonas* spp. isolates (75%; 95% CI, 58%‒92%; *n* = 18/24) were resistant to more than one antibiotic (MAR index > 0.2), with MAR indices ranging from 0.2 to 0.6 (Table 2). No *Pseudomonas* spp. isolate was susceptible to all the antibiotics tested. The distribution of the AMR findings according to their collection source is presented in Table 1. Isolates with MAR indices ≤ 0.2 may be categorized as being from low risk sources of contamination, while isolates with MAR indices > 0.2, therefore multidrug resistant (MDR), may be high‐risk sources (Krumperman, 1983). MDR strains were isolated from all collection areas where *Pseudomonas* spp. were detected. Excluding location 3, a two‐way chi‐squared analysis revealed that there was no association between the sample site and the proportion of resistant isolates, with *p* = .995.

**TABLE 2:** Distribution of zone of inhibition results (in mm) and multiple antibiotic resistance (MAR) indices (calculated as per Krumperman (1983)) for the 24 *Pseudomonas* spp. isolates obtained from wild bird feces

Isolate	Accession number	Zone of inhibition (mm)^a^					MAR Index
		MEM	FEP	CN	CIP	LEV	
*Pseudomonas* sp. strain 1.2	MN904993	25	18	22	22	21	0.6
*Pseudomonas* sp. strain 1.5	MN905002	18	15	24	21	21	0.6
*Pseudomonas* sp. strain 1.6	MN904995	26	19	19	23	26	0.4
*Pseudomonas fulva* strain 2.12	MN904979	19	11	19	21	20	0.6
*Pseudomonas* sp. strain 2.13	MN904985	30	15	26	31	23	0.2
*Pseudomonas koreensis* strain 2.16	MN904983	32	13	25	25	23	0.4
*Pseudomonas* sp. strain 2.19	MN904982	26	15	23	25	24	0.4
*Pseudomonas koreensis* strain 4.1	MN904984	26	19	22	25	22	0.4
*Pseudomonas koreensis* strain 4.9	MN904981	31	17	17	25	25	0.4
*Pseudomonas koreensis* strain 4.11	MN904997	29	20	21	25	24	0.4
*Pseudomonas* sp. strain 4.14	MN904999	22	12	17	25	24	0.4
*Pseudomonas* sp. strain 4.16	MN904987	31	20	20	26	26	0.2
*Pseudomonas koreensis* strain 4.17	MN904989	28	18	21	25	24	0.4
*Pseudomonas sp*. strain 4.19	MN904991	29	21	20	23	24	0.2
*Pseudomonas aeruginosa* strain 4.20	MN905001	36	11	26	21	19	0.6
*Pseudomonas koreensis* strain 4.23	MN904992	28	21	20	25	25	0.2
*Pseudomonas koreensis* strain 4.30	MN904994	13	21	11	20	25	0.6
*Pseudomonas* sp. strain 4.39	MN905000	28	12	18	25	26	0.4
*Pseudomonas koreensis* strain 4.47	MN904988	29	21	21	21	25	0.2
*Pseudomonas koreensis* strain 5.3	MN904986	30	19	22	26	22	0.2
*Pseudomonas* sp. strain 5.14	MN904996	26	20	21	24	24	0.4
*Pseudomonas* sp. strain 5.15	MN904998	18	20	13	24	24	0.6
*Pseudomonas fluorescens* strain 5.16	MN904990	29	17	21	25	24	0.4
*Pseudomonas* sp. strain 6.3	MN904980	20	13	20	23	18	0.6

Note

The antibiotics tested by disk diffusion method were meropenem (MEM, 10 μg), cefepime (FEP, 30 μg), gentamicin (CN, 30 μg), ciprofloxacin (CIP, 5 μg), and levofloxacin (LEV, 5 μg). The isolates' title is constituted of two components, the first number represents the sample collection location, and the second represents the temporal order of collection (for instance, strain 1.2 was the second to be collected from sample collection location 1). The cells have been colored red and green to demonstrate the isolates' antibiotic resistance and susceptibility, respectively.

^a^ Breakpoints for the tested antibiotics are as follows (The European Committee on Antimicrobial Susceptibility Testing, 2019): MEM: resistant <18 mm; FEP: resistant <21 mm; CN: resistant <15 mm; CIP: resistant <26 mm; LEV: resistant <22 mm.

There was a significant difference between the number of resistant isolates found for each of the five tested antibiotics, indicated by chi‐squared analysis, with *p* < .001. The highest prevalence of resistance within the 24 *Pseudomonas* spp. isolates was found toward ciprofloxacin (*n* = 21; 87.5%; 95% CI, 74.3%‒100%) and cefepime (*n* = 20; 83.3%; 95% CI, 68.4%‒98.2%). The lowest prevalence of resistance among the 24 *Pseudomonas* spp. isolates was found toward meropenem (*n* = 1; 4.2%; 95% CI, 0%‒12.2%), gentamicin (*n* = 2; 8.3%; 95% CI, 0%‒8.3%), and levofloxacin (*n* = 5; 20.8%; 95% CI, 4.6%‒37.1%).

## 4 DISCUSSION

Wildlife, including birds, represent an area of AMR surveillance studies that would benefit from further research due to the potential risk to humans, animals, and the environment (Hernando‐Amado et al., 2019; Pornsukarom & Thakur, 2017). In the present study, 115 bird fecal samples were collected from six locations along the River Cam in Cambridge, England. The prevalence of 0 *Pseudomonas* spp. in this study was determined to be 20.9% (*n* = 24/115; 95% CI, 12.6%‒29.2%), in contrast to previous studies where prevalence rates ranged from 2% to 10% (Janiga et al., 2007; Vidal et al., 2017). Reports of higher rates are normally associated with disease outbreaks or densely populated areas (Saleh et al., 2019; Sela et al., 2007), which is not the case in the present study. Phylogenetically, the *Pseudomonas* spp. isolates' sequences clustered closely suggesting genetic relatedness; however, there was not an apparent relationship with their AMR profiles (Figure 2).

Furthermore, the high proportion of MDR (75%; 95% CI, 58%‒92%) and absence of pansusceptibility found among the 24 *Pseudomonas* spp. isolates highlight a need for continuous surveillance efforts in order to preserve ecosystem health. The present analysis demonstrates that the resistance is widespread, and not limited to isolated locations (Table 1), as determined by the nonstatistically significant relationship between sample collection site and prevalence of resistance (*p* = .995). Collectively, these findings suggest that the nonepizootic prevalence of *Pseudomonas* spp. in wild birds around urban areas is higher than previously reported. To the authors' knowledge, data on the AMR and MDR profiles of *Pseudomonas* spp. in wild birds, outside outbreak circumstances, are limited, even if the possibility of transmission to and from other species, including humans, is a concern that should warrant more attention.

*Pseudomonas* spp. and, in particular, *P. aeruginosa*, pose a serious therapeutic challenge for treatment due to their ability to develop resistance to multiple antimicrobial classes (Lister et al., 2009; Public Health England, 2021), as supported by this present study. Here, the prevalence of resistance was highest toward ciprofloxacin (*n* = 21/24; 87.5%; 95% CI, 74.3%‒100%), a widely prescribed fluoroquinolone that is part of the WHO's Essential Medicine List (Sharland et al., 2018). Fluoroquinolones have a favorable pharmacokinetic and pharmacodynamic profile that makes them first‐line choice in the treatment of several community‐acquired and nosocomial infections, including *P. aeruginosa*. With the widespread use of the drug, resistance has emerged and continues to rapidly escalate (Lister et al., 2009; Magiorakos et al., 2012).

Importantly, this study also reported that the lowest prevalence of resistance was found toward meropenem, a critically important member of the carbapenem antibiotic class. Meropenem is a last‐line antipseudomonal that is strictly reserved to human medicine in order to preserve its effectiveness. However, the increasing usage of carbapenems in hospitals worldwide exerts a selective pressure that promotes the emergence of resistant *Pseudomonas* spp. clones in both clinical and community settings. Since susceptibility to this class of antibiotics is often not assessed or reported in animal studies due to their restricted use, this study has purposefully included a carbapenem to evaluate potential collateral consequences of human antibiotic usage in animal *Pseudomonas* spp. strains. The presence of resistance to meropenem only in a single isolate (of 24 collected in this study), suggests that meropenem‐resistant *Pseudomonas* spp. strains are not prevalent in this wild bird population. Nonetheless, one must still consider that several of the *Pseudomonas* spp. isolates were MDR, demonstrating that these bacteria may harbor different resistance mechanisms, posing a potential risk for all types of resistance, including carbapenems (Cazares et al., 2020; Lister et al., 2009).

Identifying these resistance mechanisms is key to strengthening the current understanding of evolutionary selective pressures and the potential AMR circulation and spillover routes (whole‐genome sequencing is currently ongoing; unpublished data). Alongside other studies, the present findings highlight the potential that wild birds may be one of the vectors contributing to the widespread dissemination of the bacterial pangenome, including AMR genes (Hernando‐Amado et al., 2019; Pornsukarom & Thakur, 2017).

While the identification of bacterial presence is important, the characterization of the isolates' source is also essential to assess the potential impact on the ecosystem. In the current study, the samples were collected from six urban aquatic locations along the River Cam. The River Cam runs through central Cambridge, England, and is extensively used for recreational activities both in and on the water (e.g., swimming and boating). The sampling locations selected for the study are varied and in proximity to key areas such as country parks, rowing clubs, and nature reserves, all of which are visited daily by many people and animals alike. The analysis of MAR indices suggests that some of these locations are higher risk sources of contamination than others (Table 1); however, this study did not find a statistically significant association (*p* = .995). Conversely, the prevalence of *Pseudomonas* spp. was found to differ significantly between the sampled locations (*p* < .05). This was most likely a result of the varied number of samples collected from each location (Table 1), which introduced a potentially confounding variable to the study. For instance, as a result of fieldwork limitations and bird behavior, only 6 swabs were collected from location 3 and no *Pseudomonas* spp. isolates were obtained. Additionally, several more samples were collected from other areas with more vegetation and human activity that encouraged foraging behaviors (e.g., picnicking and food littering). Nonetheless, this is a retrospective observation made by the authors and was not subject of direct analysis in this study.

From a wider, yet relevant, perspective, future work needs to focus not only on collecting/collating microbiological surveillance data from wild birds, but also on analysis, reporting, and dissemination of these data. Importantly, there must be a dedicated effort to devise interventions that are mindful of the entire ecosystem so that they can comprehensively inform the policymaking process (Wellcome Trust et al., 2018; O'Neill, 2016). For instance, as suggested in this paper, some of the drivers of AMR to wildlife may be anthropogenic; thus, reasonable interventions could include efforts to improve antimicrobial stewardship and policies regarding waste management.

## 5 CONCLUSION

In conclusion, this study provides useful information regarding the natural prevalence of MDR *Pseudomonas* spp. in wild bird fecal samples collected around the River Cam in Cambridge, England. The results have highlighted that wild birds could act as potential MDR bacterial reservoirs in areas where spillover of AMR to humans, other animals, or the environment could occur. Nevertheless, as described throughout this manuscript, determining the source and directionality of AMR dissemination is challenging which may, to a certain extent, explain why the currently available evidence is limited and/or contradictory. It is our view that in order to overcome this, future work needs to be multidisciplinary and comprehensive of the whole ecosystem, including the often forgotten wildlife fauna. The findings in this study are significant and highlight the need for a One Health Approach to tackle AMR, including increased, ongoing, nonepizootic surveillance to prevent the spread and minimize potential risks to humans, animals, and the environment.

## CONFLICT OF INTEREST

The authors have no conflicts of interest to declare.

## AUTHOR CONTRIBUTIONS

**Joana G. C. Rodrigues:** Conceptualization (equal); Data curation (equal); Formal analysis (equal); Investigation (equal); Methodology (equal); Resources (lead); Validation (equal); Visualization (equal); Writing‐original draft (lead); Writing‐review & editing (equal). **Harisree P. Nair:** Data curation (equal); Formal analysis (equal); Investigation (equal); Methodology (supporting); Software (lead); Validation (equal); Visualization (equal); Writing‐original draft (supporting); Writing‐review & editing (equal). **Christopher O'Kane:** Conceptualization (supporting); Formal analysis (equal); Funding acquisition (supporting); Methodology (equal); Supervision (supporting); Writing‐original draft (supporting); Writing‐review & editing (supporting). **Caray A. Walker:** Conceptualization (equal); Formal analysis (equal); Funding acquisition (lead); Project administration (lead); Resources (supporting); Supervision (lead); Writing‐original draft (supporting); Writing‐review & editing (equal).

## Data Availability

The partial 16S rRNA gene sequences were submitted to GenBank with accession numbers MN904979 to MN905002.
